# Critical analysis on the use of cholecalciferol as a COVID-19 intervention: a narrative review

**DOI:** 10.1590/1516-3180.2020.0532.02112020

**Published:** 2020-12-24

**Authors:** Stephanye Carolyne Christino Chagas, Francisca Sueli Monte Moreira, Irla Carla França Barbosa, Osvaldo de Sousa Leal, Leila Bastos Leal, Davi Pereira de Santana

**Affiliations:** I MSc. Pharmacist and Doctoral Student, Department of Pharmaceutical Sciences, Universidade Federal de Pernambuco (UFPE), Recife (PE), Brazil.; II PhD. Pharmacist and Adjunct Professor, Department of Pharmaceutical Sciences, Universidade Federal de Pernambuco (UFPE), Recife (PE), Brazil.; III MSc. Pharmacist and Doctoral Student, Department of Pharmaceutical Sciences, Universidade Federal de Pernambuco (UFPE), Recife (PE), Brazil.; IV MD, MSc. Family Health Doctor, Health Sciences and Sports Center, Universidade Federal do Acre (UFAC), Rio Branco (AC), Brazil.; V MSc, PhD. Pharmacist and Associated Professor, Department of Pharmaceutical Sciences, Universidade Federal de Pernambuco (UFPE), Recife (PE), Brazil.; VI MSc, PhD. Pharmacist and Full Professor, Department of Pharmaceutical Sciences, Universidade Federal de Pernambuco (UFPE), Recife (PE), Brazil.

**Keywords:** Vitamin D, Cholecalciferol, COVID-19 [supplementary concept], Severe acute respiratory syndrome coronavirus 2 [supplementary concept], Coronavirus, SARS-CoV-2, Viral infection, Prevention, Treatment.

## Abstract

**BACKGROUND:**

The World Health Organization has declared that a pandemic situation exists in relation to the disease caused by the new coronavirus, COVID-19. So far, the absence of a vaccine against the new coronavirus has led people worldwide to seek various therapeutic alternatives, including use of cholecalciferol.

**DESIGN AND SETTING:**

Narrative review developed by a research group at a public university in Recife (PE), Brazil.

**METHODS:**

We searched the literature on the use of cholecalciferol for prevention or treatment of COVID-19, using the MEDLINE and LILACS databases, with the keywords “vitamin D”, “cholecalciferol”, “SARS-CoV-2”, “COVID-19” and “coronavirus”, from January 1, 2020, to June 10, 2020. Narrative reviews, cohort studies and ecological studies were selected.

**RESULTS:**

We retrieved 32 references, of which 8 were considered eligible for intensive review and critical analysis. These comprised five narrative reviews, two observational studies and one protocol proposal. Most of the studies selected reported positive effects from use of vitamin D for prevention or treatment of COVID-19. However, there was little quantitative data to assess the real impact of using this vitamin as an intervention against this disease.

**CONCLUSIONS:**

Current studies on vitamin D used for purposes other than bone health promotion cannot be taken as support to justify its use in a disease as recent as COVID-19. Studies of greater robustness, with higher levels of clinical evidence, need to be conducted. Rational use of this vitamin needs to be ensured, thereby minimizing the impacts on the patient and the public healthcare system.

## INTRODUCTION

In December 2019, a disease caused by a new coronavirus, COVID-19, caused an epidemic in China and then spread rapidly around the world.^1^ The clinical spectrum of the disease caused by the new coronavirus is broad, covering asymptomatic infection, mild infection of the upper respiratory tract and severe pneumonia with respiratory failure. COVID-19 has led many patients to require hospitalization and semi-intensive or intensive care.^2^^,^^3^ Most complications and deaths have been reported among elderly patients with evidence of underlying diseases, such as cardiovascular, lung or kidney diseases, or cancer.^1^

So far, the absence of a vaccine for the new coronavirus has led people around the world to seek a variety of therapeutic alternatives. Consequently, sales of several drugs that have not been proven to be effective for treating COVID-19 have increased. In Brazil, one of the drugs that have contributed to this statistic was cholecalciferol, also known as vitamin D3. According to data released by the Brazilian Federal Pharmacy Council, the sale of vitamin D grew by about 35% in the first months of 2020, compared with 2019.^4^ One of the reasons for this increase is that some reports correlating use of cholecalciferol with an improvement in the immune response and reduced risk of respiratory tract infections have been published.^5^^,^^6^^,^^7^^,^^8^^,^^9^ However, it needs to be emphasized that these associations have so far only been reported in observational studies and have not been confirmed through controlled clinical studies.^10^

The role of vitamin D in promoting bone health is now well established. Nonetheless, the amounts of supplemental vitamin D to be use remain a subject of constant debate in the 21^st^ century.^11^ However, use of cholecalciferol to treat a disease as recent as COVID-19, which is not supported by randomized clinical studies, inevitably falls into the category of irrational use. Irrational use of medicines is a matter of general concern for healthcare professionals, institutions and authorities. This relates not to the intrinsic risk of the product but, rather, reflects flaws or errors within the process of using it that put users’ safety at risk. Incidents with medications can result in physical, social or psychological damage to the patient, as well as contributing to longer hospital stay, which results in cost. These considerations also gain much greater weight in the context of the current pandemic.^12^^,^^13^

Some studies have investigated the use of cholecalciferol as a therapeutic alternative for prevention or treatment of COVID-19, but no reviews focusing on critical analysis of this use have yet been conducted.

## OBJECTIVE

The objective of the current narrative review was to evaluate the proposition of using cholecalciferol for prevention or treatment of COVID-19 and the resulting implications.

## METHODS

We conducted a review of the literature considering the period from January 1, 2020, to June 10, 2020. We used the MEDLINE database (via PubMed) and LILACS (via Virtual Health Library) to identify relevant articles from the starting point of a structured question, created in accordance with the acronym PICO (Population, Intervention, Comparator and Outcomes). The population was defined as “patients with a confirmed or probable diagnosis of COVID-19 infection” and the intervention considered was “vitamin D”.

Because the analysis sought to find evidence about the use of a drug that would contribute to the clinical condition of the disease caused by the new coronavirus, terms that specified comparators, outcomes and types of study were not used. Different combinations of keywords and MeSH terms were used as search strategies to ensure a broad search strategy: “vitamin D”, “cholecalciferol”, “SARS-CoV-2”, “COVID-19” and “coronavirus”.

Firstly, the titles and abstracts of the references identified through the search strategy were screened, so that potentially eligible studies were preselected. Studies that were human trials, in English or Portuguese, in which immunological parameters in response to the viral infection caused by the new coronavirus were observed, were considered eligible. Animal studies, studies in languages other than Portuguese or English, letters, comments, reports, technical notes and editorial notes were excluded.

The articles thus selected were read in full, independently, by two authors. In cases of disagreement, a third reviewer was consulted. The following data were extracted: author, year of publication, country, study design, age of subjects (years on average), type of coronavirus, sample size, proportion of men (%), funding sources, intervention, comparator, outcomes (clinical, laboratory). In the second stage, the methodological quality and the risk of bias in the text were fully assessed using the Joanna Briggs and STROBE (Strengthening the Reporting of Observational Studies in Epidemiology) tools.^14^^,^^15^

## RESULTS

From the search in the databases, 32 references were identified. After screening the titles and abstracts, eight studies were considered eligible for critical analysis. The search in LILACS using the keywords led to four results concerning the specific topic of interest, but these were excluded in accordance with the eligibility criteria, as described in Table 1.

**Table 1.** Search of the literature in medical databases, with search strategies used for each database and number of articles extracted

Database	Research strategies	Articles found
MEDLINE (via PubMed)	(Cholecalciferol) AND (coronavirus) OR (COVID-19) OR (SARS-COV2).	8
	(Vitamin D) AND (coronavirus) OR (COVID-19) OR (SARS-COV2).	20
LILACS (via Virtual Health Library)	(Cholecalciferol) AND (coronavirus) OR (COVID-19) OR (SARS-COV2).	0
	(Vitamin D) AND (coronavirus) OR (COVID-19) OR (SARS-COV2).	4

**Figure 1.**
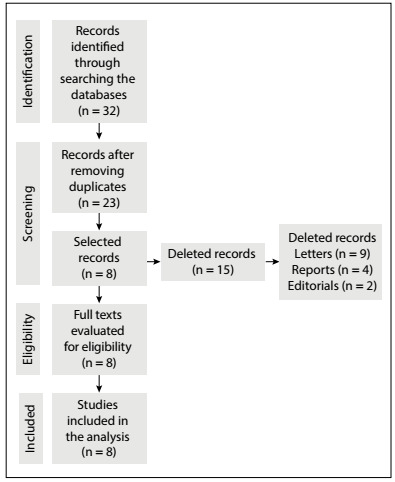
Flow diagram of the study selection process.

The article selection process is detailed in Figure 1. In this process, out of the 32 records identified, only 23 remained after removing duplicates and, of these, only eight were selected as eligible, while the other 15 references were excluded through the criteria determined in the methodology, since they were letters, reports or editorials.

Most of the studies included in this review were narrative reviews (n = 5), and there was also one protocol proposal. For these, it was not possible to apply any tool to make an evidence-based critical evaluation. The remaining two studies were observational: one of retrospective nature and the other, an ecological cohort. Only these two studies were evaluated in relation to methodological quality and risk of bias. Despite a lack of robust quantitative data on vitamin D as an intervention against COVID-19, these two studies recommended its use.

## DISCUSSION

### Main findings

The urgent need to combat the current pandemic of the new coronavirus has led to publication of several new papers suggesting alternative means of therapeutic support, such as use of vitamin D. However, none of these studies have presented high methodological quality. The current review included eight studies, among which there were five narrative reviews, one observational study, one ecological study and one retrospective cohort study. The small number of primary studies on vitamin D for treating COVID-19 made the review difficult since narrative reviews have a high potential for bias.

### Analysis on the protocol of the observational study

The study published by Caccialanza et al. suggested that vitamin D3 could be included in a protocol for early nutritional supplementation among patients with COVID-19, outside of the intensive care unit, with the aim of preventing or limiting malnutrition.^16^ This clinical protocol was based on evidence from other studies on the use of vitamin D, added to different dietary supplements, for care relating to various viral infections. However, all the studies used in this article to justify the use of this vitamin in relation to non-skeletal clinical conditions, emphasized that the antiviral mechanism of vitamin D had not been fully established, and might not accurately represent its systemic influence. Therefore, it was concluded in those studies that there was a need to conduct well-designed clinical trials.^5^^,^^6^^,^^7^^,^^8^^,^^9^

### Critical analysis on the narrative reviews

Although the role of vitamin D used for purposes other than bone health promotion is not well established, the five narrative reviews listed in our search were based on the supposed role of this vitamin in providing immunity and/or treating viral respiratory infections, with the aim of suggesting that it might be possible to use it as an intervention tool against COVID-19.^17^^,^^18^^,^^19^^,^^20^^,^^21^

According to Iddir et al., vitamin D has the particular capacity to interfere with viral cell infection through interaction with angiotensin-converting enzyme 2 (ACE2) cell input receptors (which are responsible for allowing the virus to enter the host cell).^21^ However, some studies have suggested that vitamin D can downregulate the ACE2 receptor, thus decreasing the risk of COVID-19 infection. On the other hand, other studies have suggested that vitamin D positively regulates ACE2, which would increase the level of severe acute respiratory syndrome coronavirus 2 (SARS-CoV-2) infection, thereby indicating contradictory results with limited evidence.^22^

Additionally, the narrative review published by Zabetakis et al.^20^ also showed that the data on vitamin D and immune function is ambiguous. A meta-analysis on 25 randomized clinical trials, including 11,000 patients cited by these authors, analyzed the general protective effect of vitamin D supplementation against acute respiratory diseases. However, those trials were inconclusive. Moreover, other studies on vitamin D have not shown that this has any benefit beyond bone health promotion.^21^^,^^23^ Nonetheless, several authors have speculated that occurrences of low levels of vitamin D may play a role in enabling high incidence of COVID-19, especially because these outbreaks have occurred in winter, as exemplified in a study published by Grant et al.^17^^,^^20^

Grant et al. analyzed the role of vitamin D in reducing the risk of respiratory tract infections, through an epidemiological study on influenza and COVID-19 and how vitamin D supplementation can be a useful measure for reducing the risk. According to this narrative review, the evidence that vitamin D has a role in reducing the risk of COVID-19 includes the following facts: the outbreak was during the northern hemisphere winter, when the concentrations of 25(OH)D are at their lowest; the number of cases in the southern hemisphere near the end of its summer was low; vitamin D deficiency contributes to acute respiratory distress syndrome; and lethality rates increase with age and with chronic disease comorbidity, which are both associated with lower concentrations of 25(OH)D.^17^

Not surprisingly, it has been found that serum levels of 25(OH)D are lower in patients with moderate to severe forms of COVID-19, since the comorbidities commonly presented by these individuals (chronic diseases, inflammatory diseases, obesity and diabetes) are primarily associated with vitamin D deficiency. However, this association does not determine causality and, so far, no randomized clinical study has shown any benefit from use of vitamin D in relation to prevention or treatment of COVID-19, as has been highlighted by the Brazilian Society of Endocrinology and Metabology (SBEM).^10^

The review carried out by Grant et al.^17^ used data from many articles to address the possible extra-bone effects from use of vitamin D. However, most of these studies were observational and presented high risk of bias. Five clinical studies correlating use of vitamin D with colds and flu are mentioned in the review by Grant et al., but only two of these reported any beneficial effects.^24^^,^^25^^,^^26^^,^^27^^,^^28^ Those authors further stated that, to reduce the risk of infection, it is recommended that people at risk of influenza and/or COVID-19 should consider taking 10,000 international units (IU)/day of vitamin D3 for a few weeks to rapidly increase their concentrations of 25(OH)D, followed by 5,000 IU/day.^17^

Nevertheless, another narrative review, published by Hribar et al.,^19^ also suggested that daily supplementation of 2,000-5,000 IU/day of vitamin D3, specifically for elderly people with Parkinson’s disease, could offer additional protection against COVID-19.^19^ Grant et al. also argued that, for the treatment of people infected with COVID-19, higher doses of vitamin D 3 might be useful.^17^

Likewise, a narrative review published by Calder et al.^18^ showed, among other conclusions, that supplementation above the recommended dose, but within the safety limits for specific nutrients such as vitamins C and D was necessary in order to increase the resistance to respiratory infections, such as those caused by COVID-19.^18^

Although the authors of the narrative reviews emphasized that further studies were needed because of the current situation relating to the outbreak of COVID-19, it needs to be emphasized that, although the possible extraskeletal action of vitamin D is a topic of scientific interest, no indications for prescription of vitamin D supplementation aimed at effects beyond bone health promotion have yet been approved.^10^ Consequently, additional research, with better methodological quality and evidence, is needed before any determination can be made regarding the prophylactic or therapeutic value of vitamin D against COVID-19.^29^

### Critical analysis on the original studies

Two original studies, a cross-sectional/ecological study published by Ilie et al.^30^ and a retrospective cohort study published by D’Avolio et al.,^31^ suggested that a relationship exists between serum vitamin D levels and COVID-19 infection.

The cross-sectional/ecological study published by Ilie et al.^30^ showed that there were significant associations between vitamin D levels, the number of cases of COVID-19 and the mortality caused by this infection. These authors suggested that, in several European countries, serum vitamin D levels were strongly associated with the numbers of cases of the disease caused by the new coronavirus and that supplementation with this vitamin could protect against infection by SARS-CoV-2.^30^ However, they did not clarify the period of the year over which the serum vitamin D levels were verified in the countries evaluated. This is important because these serum levels are derived from exposure to sunlight, which is influenced by several factors such as latitude, season and local weather conditions.^32^ Furthermore, the number of cases per country is affected by the number of tests performed and also by the different measures taken to prevent spreading of the infection. Therefore, the results from this study need to be interpreted cautiously. The authors themselves emphasized that the hypothesis towards which their study pointed would need to be taken forward and investigated using study designs of greater robustness.

The retrospective cohort study published by D’Avolio et al.^31^ proposed that concentrations of 25-hydroxyvitamin D would be lower in patients with SARS-CoV-2 who were positive for C-reactive protein (CRP).^31^ The study was conducted in Switzerland, among 1,484 patients of both sexes and of ages ranging from 18 to 70 years. However, although its design presented greater strength of evidence than the studies cited above (observational study and narrative reviews), no inclusion/exclusion criteria were established among the study groups. Thus, the only variables assessed were sex and age. Consequently, the profile of the patients seen remained unclear with regard to disease severity, and the description of the study subjects did not allow verification of whether the populations were comparable. Considering that most complications and deaths due to COVID-19 have been reported among elderly patients with evidence of underlying diseases, such as cardiovascular, pulmonary or kidney disease, or cancer, the limiting criteria established in this study increased the risk of bias.^1^ Additionally, it needs to be considered that vitamin D levels may also vary according to hormonal, genetic and nutritional factors.^33^ Thus, the analysis did not allow identification of whether vitamin D deficiency was an underlying illness rather than the cause. Confounding factors would therefore possibly arise, especially due to the lack of information about the population at the baseline, which could influence the direction of the results. Although the authors considered some of these factors, they did not describe the strategies used to deal with confounding factors.

Therefore, it becomes evident that further research needs to be conducted, especially given that the amount of supplemental vitamin D to be administered continues to be a matter for debate in the 21^st^ century, even in relation to well-established diseases such as those linked to mineral metabolism. The discussion becomes even more complex with regard to a disease as recent as that caused by the new coronavirus.^11^^,^^34^

Thus, the urgent need to combat the current pandemic must not override the need to make rational use of medicines. In the case of cholecalciferol, several studies have suggested that vitamin D intoxication may occur when doses greater than 10,000 IU are administered daily, for periods lasting from several months to some years.^35^ Therefore, building up a toxic dose of vitamin D through supplementation is a real possibility. This can lead to consequences such as hypercalcemia and hypercalciuria, with consequent risks of renal failure, seizures and death.^10^^,^^36^^,^^37^

In Brazil, medications are responsible for more than 52% of intoxications in this country, and 15% to 20% of hospital budgets are spent on treatment of complications resulting from them.^38^^,^^39^ Considering that public healthcare systems worldwide are becoming depleted through the current pandemic, it is essential that events caused by irrational use of medicines should be avoided and that extrapolation of their use to form treatments for COVID-19 should be discussed better and be based on well-supported clinical studies.

### Strengths and limitations

The articles included in this review generated heterogeneous data because of the diversity in the design of the studies (five narrative reviews, two observational studies and one treatment protocol).

The main limitation of this review was the lack of tools for methodological assessment of narrative reviews. Nonetheless, these reviews were maintained in the present study in view of the scarcity of data on the use of vitamin D for treatment of COVID-19. The studies lacked high levels of scientific evidence to support their conclusions.

As far as we know, this was the first study to critically review and evaluate the use of vitamin D as an intervention tool for treatment of COVID-19. Thus, this study is of great value for safe decision-making regarding rational use of this medicine in the context of the current pandemic.

## CONCLUSIONS

In the studies included through the systematic search of this review, no robust and conclusive evidence regarding the effectiveness of the use of vitamin D for prevention or treatment of COVID-19 was identified. The studies analyzed presented limitations regarding their designs and methodologies, which implied high risk of bias.

Thus, the current studies on use of vitamin D for purposes other than bone health promotion cannot be taken as support for justifying its use in a disease as recent as COVID-19. Hence, further studies with greater robustness of clinical evidence need to be conducted. Through this, rational use of this vitamin will be ensured and the impacts on patients and the public healthcare system will be minimized.
